# ﻿A new species of *Quercus* genus (Fagaceae) from Son Tra Peninsula, Central Vietnam

**DOI:** 10.3897/phytokeys.206.85635

**Published:** 2022-08-26

**Authors:** Nguyen Van Ngoc, Hoang Thi Binh, Hoang Thanh Son, Yoshihisa Suyama, Tetsukazu Yahara

**Affiliations:** 1 Faculty of Biology, Dalat University, 01 – Phu Dong Thien Vuong, Dalat, Vietnam Dalat University Dalat Vietnam; 2 Department of Forest Phytodiversity, Silviculture Research Institute, Vietnamese Academy of Forest Sciences, Hanoi, Vietnam Silviculture Research Institute, Vietnamese Academy of Forest Sciences Hanoi Vietnam; 3 Field Science Center, Graduate School of Agricultural Science, Tohoku University, 232–3 Yomogida, Naruko-onsen, Osaki, Miyagi 989–6711, Japan Tohoku University Miyagi Japan; 4 Kyushu Open University, 744 Motooka, Fukuoka, 819–0395, Japan Kyushu Open University Fukuoka Japan

**Keywords:** Da Nang City, flora, MIG-seq, phylogeny, taxonomy

## Abstract

A new species, *Quercussontraensis* Ngoc, Binh & Son is described from Son Tra Nature Reserve, Son Tra Peninsula, Central Vietnam. We examined the morphology and constructed a highly resolved phylogeny of *Q.sontraensis* and its relatives (including *Q.langbianensis* and *Q.cambodiensis*) using Multiplex ISSR genotyping by sequencing (MIG-seq). The morphological analyses and molecular evidence support the distinction between the new species (*Q.sontraensis*) and its relatives.

## ﻿Introduction

The genus *Quercus* contains more than 500 species worldwide, amongst which about 400 species from the Americas, Europe, North Africa and Macaronesia and about 125 species were reported from Asia (Govaerts and Frodin 1998; Borazan and Babaç 2003; Hubert et al. 2014), 50 of which are distributed in Vietnam (Ho 2003; Ban 2005; Li et al. 2016; Binh et al. 2018a, b, c; Binh et al. 2021). *Quercus* species are usually trees or sometimes shrubs that mostly occur in tropical montane forests in Southeast Asia and are often dominant in temperate deciduous forests in East Asia, Europe, North America, the Mediterranean and in desert scrubs of North America (Nixon 1993; Huang et al. 1999; Phengklai 2008; Hubert et al. 2014; Valencia-A et al. 2016). The members of the genus are characterised by staminate inflorescences lax spikes or dichasia (“catkins”), carpellate flowers in stiff spikes (actually double spikes, but with reduced side flowers, leading to effective solitary flowers), subtended by an indehiscent cupule with cupule bracts being connate or forming concentric or spiral rings or free and imbricate cupule bracts; capitate or dilated stigma (Kaul 1985; Nixon 1993; Huang et al. 1999; Manos et al. 1999; Phengklai 2008).

**Figure 1.**
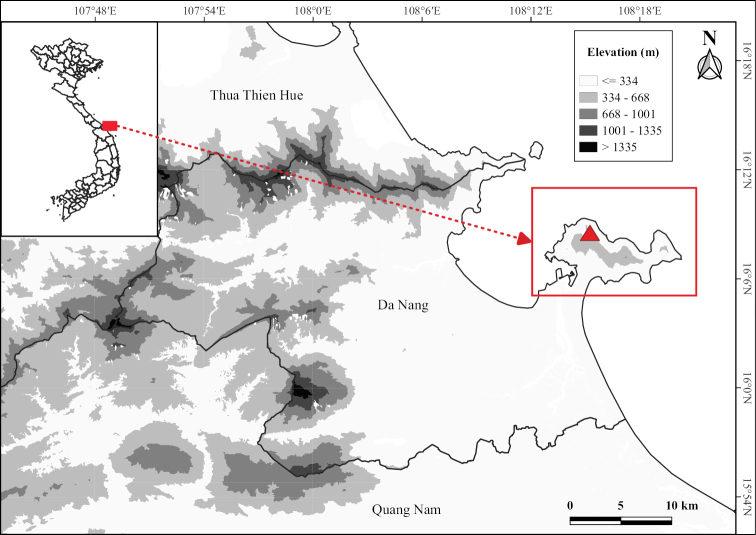
Study site: The area inside the red rectangle is Son Tra Peninsula; the red triangle is the type locality of *Quercussontraensis* Ngoc, Binh & Son.

Son Tra Nature Reserve is located on the Son Tra Peninsula of Da Nang City (Fig. 1) and was established in 1977 with a total area of 3.871 ha (Tran et al. 2019). The vegetation in the Son Tra Nature Reserve is characterised as a semi-evergreen seasonal forest (Wright 1996) with 1,032 species of vascular plants (483 genera from 145 families) recorded (Son et al. 2018).

During a field excursion to the Son Tra Nature Reserve carried out in 2016 and 2019, we collected specimens of *Quercus* from 340 m to 430 m altitude, which we were unable to allocate to a described species. Further studies, based on specimens in the herbaria such as DLU, FU, HN, P and VNM, the digitised specimen images on the website of JSTOR Global Plants and literature on related species (Camus 1936–1954; Huang et al. 1999; Ho 2003; Ban 2005; Phengklai 2008; Li et al. 2016; Binh et al. 2018a, b, c; Binh et al. 2021), showed that the samples were morphologically distinct from previously known taxa of the genus *Quercus*. In addition to using morphological evidence, the evidence from molecular analyses is helpful for delimiting species of the Fagaceae family. Specifically, Binh et al. (2018b), Ngoc et al. (2021) and Ngoc et al. (in press) successfully used molecular markers of both classic and next-generation sequencing methods to construct a highly resolved phylogenetic tree of the species of *Quercus* and *Lithocarpus*, as well as to describe new species from these genera. For the genus *Quercus*, recently, *Q.xuanlienensis* Binh, Ngoc & Bon (Binh et al. 2018a), *Q.trungkhanhensis* Binh & Ngoc (Binh et al. 2018c), *Q.baolamensis* Binh & Ngoc, *Q.bidoupensis* Binh & Ngoc and *Q.honbaensis* Binh, Tagane & Yahara (Binh et al. 2018b) have been described as new species, based on both morphological and molecular evidence.

By combining the molecular evidence and morphological observations, we describe and illustrate the unknown species from Son Tra as *Quercussontraensis* Ngoc, Binh & Son. Additionally, this study used Multiplex ISSR genotyping by sequencing (MIG-seq: Suyama and Matsuki 2015) to determine the identities and phylogenetic relationship of new species from Son Tra and its close species from Vietnam and surrounding countries.

## ﻿Materials and methods

### ﻿Plant materials

A total of fifteen samples of *Quercus* from Vietnam and Cambodia including, *Q.sontraensis* and its related species, were collected for molecular analysis. Three samples of *L.dahuoaiensis*, *L.vuquangensis* and *L.vinhensis* were included as an outgroup in the inference of the phylogenetic tree. The information on samples used for molecular analysis in this study is shown in Table 1.

**Table 1.** Sample list for MIG-seq analysis in this study.

Country	Area	Voucher	Elevation	Species
**Vietnam**	Lam Dong	V9723	1930 m	*Quercuslanata*
	Lam Dong	V3172	890 m	*Q.setulosa*
	Cao Bang	V6066	767 m	*Q.trungkhanhensis*
	Da Nang	V3156	340 m	*Q.austrocochinchinensis*
	Da Nang	V3113	1310 m	*Q.poilanei*
	Lam Dong	V10132	1630 m	*Q.poilanei*
	Lam Dong	V9884	1637 m	*Q.braianensis*
	Lam Dong	V4445	1464 m	*Q.braianensis*
	Lam Dong	V10069	1867 m	*Q.bidoupensis*
	Lam Dong	V10090	1884 m	*Q.bidoupensis*
	Lam Dong	V5537	N/A	*Q.djiringensis*
	Lam Dong	V10061	1867 m	*Q.langbianensis*
	Lam Dong	V9972	1430 m	*Q.langbianensis*
	Da Nang	QC201	340 m	*Q.sontraensis*
	Ha Tinh	V5743	1518 m	*L.vuquangensis*
	Nghe An	V3787	1062 m	*L.vinhensis*
	Lam Dong	V3194	225 m	*L.dahuoaiensis*
**Cambodia**	Bokor	C4302	844 m	*Q.cambodiensis*

In addition, the following specimen vouchers of *Q.cambodiensis*: *Poilane 215* (P [P00379257, P00379258] and NY [NY00253790]), *Poilane 270* (P [P06872434, P06872435]), *Tagane et al. C4302* (FU) and *Toyama et al. 1834* (FU), the following specimen vouchers of *Q.langbianensis*: *Chevalier 30029* (P [P00379254, P00379255, P00379256]), *Tagane et al. V4465*, *V4165*, *V4166*, *V3962* (DLU, FU) and the following specimen vouchers of *Q.sontraensis*: *Son et al. QC201* (DLU, VAFS: three duplicates), *Son H.T. QC202* (DLU, VAFS: two duplicates) and *Son H.T. QC203* (DLU, VAFS) were selected to measure the key morphological characters in the morphological analysis (a total 33 leaf of each species were measured).

### ﻿Morphological analysis

To identify new samples that were collected from Son Tra Nature Reserve (Fig. 1), we referred to taxonomic literature of the genus *Quercus* in Vietnam and its surrounding countries (Camus 1936–1954; Soepadmo 1972; Huang et al. 1999; Ho 2003; Ban 2005; Phengklai 2008; Li et al. 2016; Binh et al. 2018a, b, c). Moreover, our specimens were also examined and compared with herbarium specimens at DLU, FU, HN, P and VNM, as well as images of type specimens on virtual herbaria websites (such as JSTOR Global Plants and the Chinese Virtual Herbarium).

Additionally, to provide strong evidence for the new species, we analysed and compared several key morphological characteristics including petiole length, leaf blade length and width and leaf blade aspect ratio, based on specimens of the new species and its related species. We used ImageJ (Schneider et al. 2012) to measure the above-mentioned morphological characters.

To evaluate differences amongst species, analysis of variance (ANOVA) and Game-Howell post hoc test (Games and Howell 1976) were performed to confirm whether the differences were statistically significant (with the mean difference being significant when *p* < 0.05). The RStudio ver. 1.4.1106 (RStudio Team 2021) with ggstatsplot package (Patil 2021) and other built-in packages available in R were used to perform all statistical analyses in this study.

### ﻿DNA extraction

For DNA extraction, we used the dried leaf material and milled them into fine powder with a QIAGEN TissueLyser. Then the powder was washed three times by 1 ml buffer solution (comprising 0.1 M HEPES, pH 8.0; 2% Mercaptoethanol; 1% PVP; 0.05% Ascorbic acid) (Toyama et al. 2015). Finally, DNA was isolated from the washed powder by using the CTAB method (Doyle and Doyle 1987) with a slight modification by Toyama et al. (2015).

### ﻿Next-generation DNA sequencing

Fifteen DNA samples of eleven *Quercus* species were used to amplify thousands of short sequences by using the primers of “multiplexed ISSR genotyping by sequencing” (MIG-seq: Suyama and Matsuki 2015). We performed two steps of PCR following the protocol of Suyama and Matsuki (2015) with minor modifications as in Binh et al. (2018b). PCR products of the 2^nd^ PCR step were pooled as a single mixture library then we purified the mixture. Subsequently, we selected fragments of the size range 350–800 bp from the purified mixture by using a Pippin Prep DNA size selection system (Sage Science, Beverly, MA, USA). Then approximately 10 pM of libraries were measured by quantitative PCR (Library Quantification Kit; Clontech Laboratories, Mountain View, CA, USA) and used for sequencing in an Illumina MiSeq Sequencer (Illumina, San Diego, CA, USA) with a MiSeq Reagent Kit v.3 (150 cycles, Illumina) (Suyama and Matsuki 2015; Binh et al. 2018a, b).

### ﻿Phylogenetic tree construction

The raw data of DNA sequence were pretreated following the published protocol (Suyama and Matsuki 2015; Binh et al. 2018b; Ngoc et al. 2021) by using the trimmomatic software version 0.40 (Bolger et al. 2014). The Stacks 2.41 pipeline (Catchen et al. 2013; Rochette et al. 2019) with the parameters set as described by Takata et al. (2019) and Ngoc et al. (2021) were used for de novo SNP discovery. The Maximum Likelihood tree was constructed by using the RAxML ver. 8.2 (Stamatakis 2014), based on the genome-wide SNPs dataset with the GTR+G model as selected by jMrModeltest 2.1.10 (Darriba et al. 2012) and examined its reliability by bootstrapping using 1000 replicates.

## ﻿Results

### ﻿Morphological comparison of the new species with its close species

*Quercussontraensis* is morphologically most similar to *Q.cambodiensis* Hickel & A.Camus and *Q.langbianensis* Hickel & A.Camus. The comparison of key morphological characters amongst *Q.sontraensis*, *Q.cambodiensis* and *Q.langbianensis* are shown in Tables 2 and 3.

**Table 2.** Morphological comparison amongst *Quercussontraensis* Binh, Ngoc & Son, sp. nov., *Quercuscambodiensis* Hickel & A.Camus and *Quercuslangbianensis* Hickel & A.Camus.

Characters	*Q.sontraensis*	*Q.cambodiensis* ^(1,2,3)^	*Q.langbianensis* ^(4,5,6)^
Young shoot	Curly golden hairy	Golden tomentose	Golden tomentose
Leaf margin	Regularly and distinctly serrate on upper 1/3–1/4(–1/5)	Almost entire or with a few low teeth in upper 1/4	Distinctly serrate in upper 1/3
Length of petioles	(0.7–)1–1.5 cm	1–2.2 cm	1–2 cm
Number of secondary veins	(8–)11–14 pairs	7–11 pairs	10–12 pairs
Infructescence	Each infructescence with 1–3 acorns	Each infructescence with 4 acorns	Each infructescence with 2 acorns
Cupule shape	Bowl-shaped	Cup-shaped	Cup-shaped
Number of rings on cupule	7–8 rings	7–8 rings	6–9 rings
Margin of rings on cupule	Distinctly toothed in all rings, except two upper rings	Distinctly toothed in two lower rings	Distinctly toothed in all rings
Nut enclosure by cupule	Enclosing 1/3 of the nut	Enclosing < 1/2 of the nut	Enclosing 1/3 of the nut
Nut shape	Broadly ellipsoid	Obovoid to ellipsoid	Obovoid to ellipsoid
Base of the nut	Slightly convex	Slightly convex	Convex

^(1)^ From the material *E. Poilane 215* (P00379257) ^(2)^ From the original description in Hickel and Camus (1923)^(3)^ From the material *Tagane et al. C4302* (FU) ^(4)^ From the original description in Ann. Sci. Nat., Bot. X, 3: 382 1921 ^(5)^ From the material *Chevalier 30029* (P) ^(6)^ From the material *Tagane et al. V4166* (DLU)

According to the original description of *Q.cambodiensis*, Hickel and Camus (1923) described this species, based on two collections by Eugène Poilane (*Poilane 215* and *Poilane 270*) from Cambodia. Of which, *Poilane 215* has three duplicates in P and NY Herbaria (P00379257, P00379258 and NY 00253790) and *Poilane 270* has two duplicates in P (P06872434 and P06872435). Amongst those specimens, only *Poilane 215* [P00379257] represents the diagnostic traits of nuts and cupules. The new species is most similar to *Q.cambodiensis* in lanceolate to elliptic leaves, densely golden hairy cupules with 7–8 rings and densely golden hairy nuts, but differs from *Q.cambodiensis* in having a distinctly serrate leaf margin in upper 1/3 (vs. leaf margin almost entire or with a few low teeth in upper 1/4) and cupules with a bract margin distinctly toothed in all rings, except two upper rings (vs. distinctly toothed only in two lower rings in *Q.cambodiensis*). *Quercussontraensis* is also morphologically similar to *Q.langbianensis* Hickel & A.Camus (types: *Chevalier 30029*, P [P00379254, P00379255, P00379256]) in leaf shape, leaf margin distinctly serrate in upper 1/3 and cupules covering 1/3 of a nut. However, *Q.sontraensis* is distinct from *Q.langbianensis* in having bowl-shaped cupules (vs. cupules cup-shaped), cupule bract margin distinctly toothed in all rings, except two upper rings (vs. distinctly toothed in all rings), cupules bowl-shaped (vs. cup-shaped), nuts broadly ellipsoid (vs. obovoid to ellipsoid) and nut scar slightly convex (vs. more strongly convex). These general differences are shown in Table 2.

**Figure 2.**
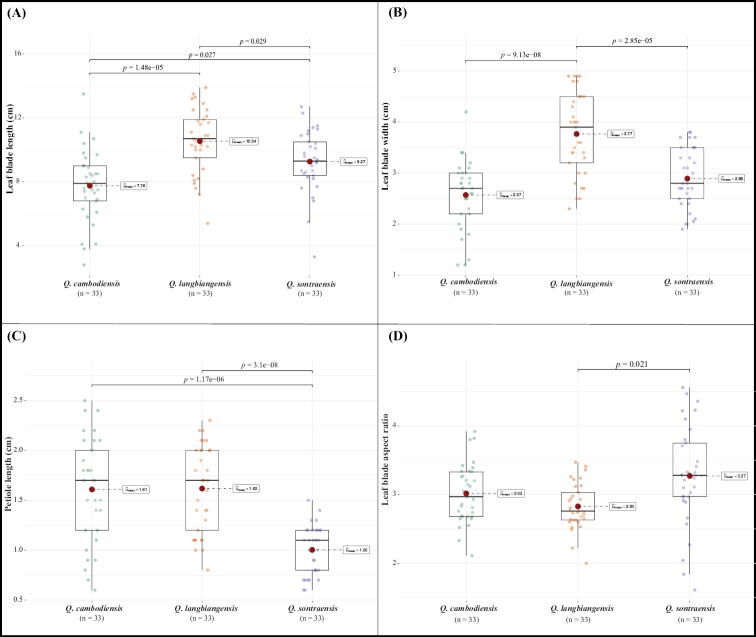
Morphological comparison of *Q.sontraensis* and its related species **A** box plot of leaf blade length **B** box plot of leaf blade width **C** box plot of petiole length **D** box plot of leaf blade aspect ratio. Red dots in the box plots indicate the mean value. Significant differences level (p) resulted from a Games-Howell post-hoc test (Comparison shown: only significant).

The morphological comparison of leaf traits amongst *Q.sontraensis* and *Q.cambodiensis* and *Q.langbianensis* (Table 3, Fig. 2) shows that the leaf blade length and width are significantly longer and relatively broader than those of *Q.cambodiensis* (9.27 ± 1.93 cm vs. 7.76 ± 2.25 cm and 2.89 ± 0.59 cm vs. 2.57 ± 0.66 cm, respectively), while significantly shorter and smaller leaf blade length and width compared to *Q.langbianensis* (9.27 ± 1.93 cm vs. 10.54 ± 2.02 cm and 2.89 ± 0.59 cm vs. 3.77 ± 0.80 cm, respectively). In addition, the new species has, on average, a significantly shorter petiole length than that of *Q.cambodiensis* and *Q.langianensis* (1.0 ± 0.24 cm vs. 1.61 ± 0.51 cm and 1.62 ± 0.43 cm, respectively). Besides, the leaf blade aspect ratio of the new species is significantly greater than that of *Q.langbianensis* (3.27 ± 0.73 cm in *Q.sontraensis* vs. 2.83 ± 0.33 cm in *Q.langbianensis*), but there are no significant differences in the leaf blade aspect ratio between *Q.sontraensis* and *Q.cambodiensis* (Table 3, Fig. 2).

**Table 3.** The comparisons of mean (X) and standard deviation (SD) value of the leaf blade amongst *Quercussontraensis*, *Q.cambodiensis* and *Q.langbianensis*. ^1^Derived from type specimens, ^2^Derived from our collections, n = number of leaves measured in this study.

Parameters	*Q.sontraensis* ^1^			*Q.langbianensis* ^1,2^			*Q.cambodiensis* ^1,2^		
	X	SD	n	X	SD	n	X	SD	n
Leaf blade length (cm)	9.27	1.93	33	10.54	2.02	33	7.76	2.25	33
Leaf blade width (cm)	2.89	0.59	33	3.77	0.80	33	2.57	0.66	33
Petiole length (cm)	1.0	0.24	33	1.62	0.43	33	1.61	0.51	33
Leaf blade aspect ratio	3.27	0.73	33	2.83	0.33	33	3.02	0.43	33

type specimens, ^2^Derived from our collections, n = number of leaves measured in this study.

### ﻿Phylogenetic tree using MIG-seq

**Figure 3.**
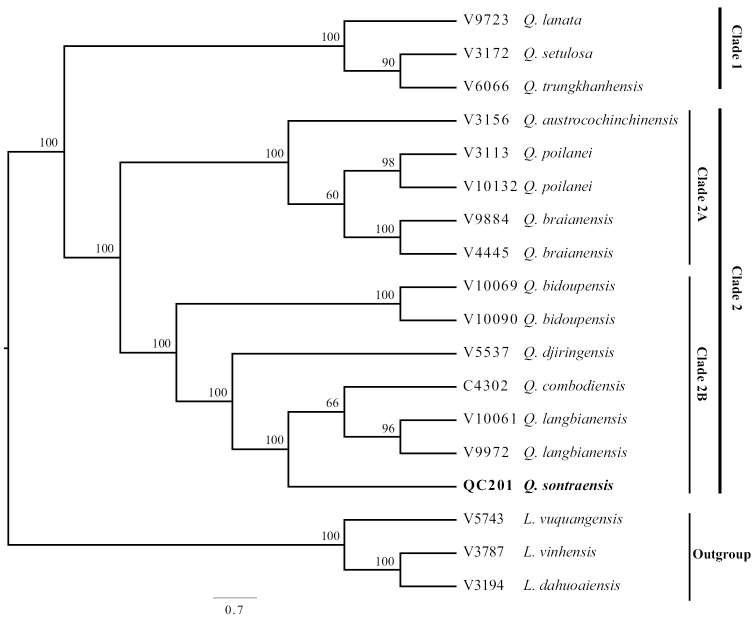
The phylogenetic relationship of the new species (Bold) with its related species, based on SNPs dataset from MIG-seq.

The Maximum Likelihood tree, based on MIG-seq data for 15 samples of *Quercus*, strongly supports two major clades (except outgroup: *Lithocarpusvuquangensis*, *L.vinhensis and L.dahuoaiensis*) consisting of clade 1 and clade 2, with 100% bootstrap value (Fig. 3). Clade 1 consists of three species of section Ilex of subgenus Cerris with scale-cup oaks (Denk et al. 2017) (including *Q.lanata*, *Q.setulosa and Q.trungkhanhensis*) and clade 2 including 12 samples of eight species of section Cyclobalanopsis of subgenus Cerris with concentric lamellae-cup (Denk et al. 2017) (including *Q.austrocochinchinensis*, *Q.poilanei*, *Q.braianensis*, *Q.bidoupensis*, *Q.djiringensis*, *Q.cambodiensis*, *Q.langbianensis* and the new species as “*Q.sontraensis*”). The new species is placed in a highly supported monophyletic group (clade 2B with 100% bootstrap value) which included *Q.bidoupensis*, *Q.djiringensis*, *Q.cambodiensis* and *Q.langbianensis*. Amongst those five species of clade 2B, *Q.sontraensis* is placed together with *Q.cambodiensis* and *Q.langbiangensis* with 100% bootstrap value. While the two accessions of *Q.langbianensis* are clustering together with 96% bootstrap support, a sister relationship of *Q.langbianensis* and *Q.cambodiensis* was only supported with 66%.

## ﻿Discussion

The morphological examination and the MIG-seq tree support *Quercussontraensis* as being closely related to *Q.langbiangensis* and *Q.cambodiensis*. Morphological differentiation of leaf characters is moderate and mainly shown through statistical measures (Fig. 2, Table 3), while simple comparisons might not be too helpful (Table 2). Cupule and nut characters seem to provide good qualitative characters to recognise *Q.sontraensis* (Table 2). In addition, *Q.sontraensis* was found at different elevations in Vietnam and Cambodia, respectively. While *Q.sontraensis* was collected at a lower elevation, around 340 m, the two other species were recorded at higher elevations, > 800 m. The combination of ecological differences and morphological and phylogenetic analysis provides good evidence for recognising the new species in this study.

### ﻿Taxonomic treatments

#### ﻿ Quercus sontraensis

Taxon classificationPlantaeFagalesFagaceae

Ngoc, H.T.Binh & Son sp. nov.

96F58713-9F28-5FF3-BB78-857B00B7396A

urn:lsid:ipni.org:names:77303991-1

Fig. 4

##### Diagnosis.

*Quercussontraensis* is morphologically similar to *Q.cambodiensis* and *Q.langbianensis* in leaf shape (lanceolate to elliptic), cupules with 6–9 rings and cupule enclosing less than ½ of the nut. However, *Q.sontraensis* is distinguished from *Q.cambodiensis* by its leaf margin regularly and distinctly serrate on the upper 1/3–1/4 (–1/5) of the lamina (vs. almost entire or with a few low teeth in upper 1/4), bowl-shaped cupule (vs. cup-shaped), cupule bract margin distinctly toothed in all rings, except two upper rings (vs. entire, except distinctly toothed in two lower rings) and differs from *Q.langbianensis* in having bowl-shaped cupule (vs. cup-shaped), cupule bract margin distinctly toothed in all rings, except two upper rings (vs. distinctly toothed in all rings), broadly ellipsoid nut (vs. obovoid to ellipsoid) and slightly convex nut scar (vs. convex).

**Figure 4.**
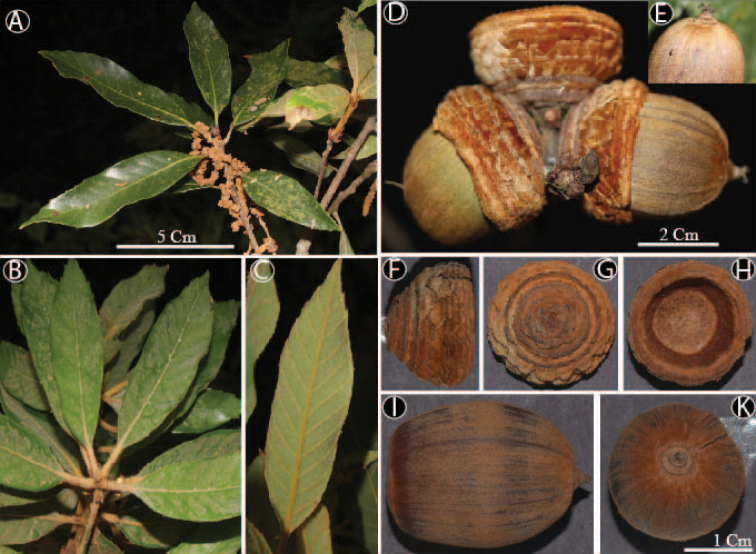
*Quercussontraensis* Binh, Ngoc & Son **A** leafy twig with male inflorescences **B, C** young leaves covered with densely golden hairs **D** mature fruits **E** stylopodium **F, G** side and base of the cupule **H** inside of the cupule **I** nut (lateral view) **K** nut (top view). Materials from *Son et al. QC201*.

##### Type.

Vietnam. Da Nang City, Son Tra Peninsula, Son Tra Nature Reserve, in evergreen forest, alt. 340 m, 16°08'22.90"N, 108°15'28.85"E, 9 October 2016, *Son H.T.*, *Binh H.T.*, *Ngoc N.V. QC201* (holotype DLU!, isotypes HN!, VNM!, VAFS!).

##### Description.

Tree, 12 m tall. Terminal and lateral buds ovoid, 3–5 mm long, 1.5–3 mm in diam., scales in 3–5 rows, imbricate, ovate-triangular, ca. 1.5 × 2 mm, apex obtuse, margin yellowish-brown ciliate, appressed hairy on both surfaces. Young twigs greyish-brown, 1.5–2 mm in diam., densely curly golden hairy, sometimes sulcate, old twigs greyish-brown, glabrous, lenticellate. Leaves alternate; blades elliptic to elliptic-lanceolate, 7–12.5 × 2.2–3.5 cm, acuminate or sometimes acute at apex, cuneate at base, margin regularly and distinctly serrate in the upper 1/3–1/4 (–1/5), densely golden hair on both surfaces when young, glabrescent, midrib sunken adaxially, prominent abaxially, lateral veins (8–)11–14 pairs, straight and running into the teeth of margin, slightly sunken adaxially, prominent abaxially, at an angle of 45–50(–52) degrees from midrib, tertiary veins scalariform, faint on the upper surfaces and conspicuous on the lower surfaces; petioles (0.7–)1–1.5 cm long, densely curly golden hairy when young, soon glabrous. Male inflorescence 7–9 cm long. Female inflorescence 2.5–3.5 cm long, female flowers solitary. Infructescences axillary or terminal, erect, rachis 0.5–0.8 (–1.2) cm long, 3–4 (–6) mm in diam., densely golden hairy. Fruits 1–3, 3–3.3 cm long (including cupule), sessile; cupules bowl-shaped, 1.2–1.7 (–1.9) cm long, 1.3–2.2 cm in diam., enclosing 1/3 of the nut when mature, both outside and inside covered with densely appressed yellowish-brown hairs, wall ca. 1.5–3 mm thick, comprising scales, scales arranged in 7–8 rings, margin of the ring distinctly toothed in all rings, except two upper rings; nut broadly ellipsoid 2.3–2.6 cm long, 1.7–2.0 cm in diam., densely golden hairy, apex nearly flat, densely appressed curly golden hairs around stylopodia, stylopodia up to 1.5 mm long, basal scar 1.1–1.3 cm in diam., slightly convex, glabrous.

##### Distribution.

Vietnam. Da Nang City, Son Tra Nature Reserve.

##### Etymology.

The specific epithet is derived from the district name of the type locality, Son Tra Nature Reserve, Son Tra Peninsula, Da Nang City, Central Vietnam.

##### Vernacular name.

Sồi Sơn Trà (suggested here).

##### Phenology.

Flowering from January to March, fruiting specimens were collected from September to October.

##### Additional specimens examined.

Vietnam. Da Nang City, Son Tra Peninsula, Son Tra Nature Reserve, in evergreen forest, 385 m elev., 16°07'41.7"N, 108°15'55.7"E, 20 September 2019, *Son H.T. QC202* [fr.] (DLU!, VAFS!); ibid., 428 m elev., 16°07'00.3"N, 108°17'40.5"E, 20 September 2019, *Son H.T. QC203* [fl.] (DLU!, VAFS!).

##### Preliminary conservation status.

During our floristic survey inside the protected area of Son Tra Nature Reserve, less than 10 mature individuals of *Quercussontraensis* were found in the evergreen forest, from 340 to 430 m altitude. According to the criteria established by the IUCN Red List (IUCN 2019), *Q.sontraensis* is qualified as Critically Endangered (CR), based on the extent of occurrence (EOO 1.47 km^2^) and area of occupancy (AOO 12.0 km^2^) [CR B1ab(i,ii,iii) 1 B2ab(i,ii,iii)].

## Supplementary Material

XML Treatment for Quercus sontraensis
